# Endogenous complement-activating IgM is not required for primary antibody responses but promotes plasma cell differentiation and secondary antibody responses to a large particulate antigen in mice

**DOI:** 10.3389/fimmu.2023.1323969

**Published:** 2024-01-08

**Authors:** Anna-Karin E. Palm, Annika Westin, Diyar Ayranci, Birgitta Heyman

**Affiliations:** Department of Medical Biochemistry and Microbiology, Uppsala University, Uppsala, Sweden

**Keywords:** complement, IgM, c1q, IgM-mutation, antibody feedback regulation

## Abstract

Lack of complement factor C1q of the classical pathway results in severely impaired primary antibody responses. This is a paradox because antibodies, especially IgM, are the most efficient activators of the classical pathway and very little specific IgM will be present at priming. A possible explanation would be that natural IgM, binding with low affinity to the antigen, may suffice to activate complement. In support of this, mice lacking secretory IgM have an impaired antibody response, which can be rescued by transfer of non-immune IgM. Moreover, passive administration of specific IgM together with antigen enhances the antibody response in a complement-dependent fashion. To test the idea, we have used a knock-in mouse strain (Cμ13) carrying a point mutation in the IgM heavy chain, rendering the IgM unable to activate complement. Mutant mice backcrossed to BALB/c or C57BL/6 background were primed and boosted with a low dose of sheep red blood cells. Confirming earlier data, no impairment in early, primary IgM- or IgG-responses were seen in either of the Cμ13 strains. However, in one of the mutant strains, late primary IgG responses were impaired. A more pronounced effect was observed after boost, when the IgG response, the number of germinal center B cells and antibody secreting cells as well as the opsonization of antigen were impaired in mutant mice. We conclude that complement activation by natural IgM cannot explain the role of C1q in primary antibody responses, but that endogenous, specific, wildtype IgM generated after immunization feedback-enhances the response to a booster dose of antigen. Importantly, this mechanism can only partially explain the role of complement in the generation of antibody responses because the IgG response was much lower in C3- or complement receptor 1 and 2-deficient mice than in Cμ13 mice.

## Introduction

The complement system consists of a number of proteins and cell surface receptors that are involved in many adaptive and innate immune functions such as inflammation, opsonization, cytotoxicity and immune regulation. The importance of complement for the generation of normal antibody responses was first discovered almost 50 years ago by Pepys (1) whose studies demonstrated that mice, transiently depleted of 95% of their C3 by administration of cobra venom factor, had poor antibody responses to sheep red blood cells (SRBC). This finding has been confirmed and extended, e.g. using gene targeted mice and animals with hereditary deficiencies of a specific complement component (reviewed in (2)). Lack of C1q (3, 4), C2 (5), C3 (6, 7), C4 (6, 8, 9), or complement receptors 1/2 (CR1/CR2) (4, 10–13) leads to impaired antibody responses. Because the phenotype in animals lacking any of these components is similar, it is assumed that the major role of C1q, C2, C3, and C4 in antibody responses is to generate the C3 split products constituting ligands for CR1/CR2. Mice lacking component C5 of the terminal pathway (14), or factor B of the alternative pathway (15, 16), have normal antibody responses whereas mice deficient in mannan-binding lectin (MBL) of the lectin pathway may exhibit normal, moderately lower or moderately higher antibody responses (17–20). Importantly, the reduced antibody response in MBL-deficient mice is never as strong as that seen in C1q-deficient mice (3, 4). Altogether, these findings suggest that the classical pathway, and not the alternative or lectin pathways, plays a major role in the generation of antibody responses. However, this appears to be a paradox because also primary antibody responses are impaired in complement-deficient (1, 4, 6, 9, 21) or CR1/CR2-deficient (6, 12) animals. The classical pathway is primarily activated by antibodies, yet, naturally, very low amounts of specific antibodies would be present at the start of a primary antibody response to any given antigen. A possible solution to this enigma presented itself when it was shown that mice lacking secretory IgM have an impaired antibody response which could be reconstituted with IgM from naive mice (22), suggesting that binding of natural, low-avidity IgM to an antigen would suffice for rapid activation of complement during an early immune response. The idea agreed well with earlier data showing that passive administration of specific IgM together with SRBC resulted in enhancement of the antibody responses (23, 24) and that this enhancement was dependent on the ability of IgM to activate complement (25, 26).

A decade ago, we generated the Cµ13 mouse strain to formally test the hypothesis that activation of the classical complement pathway by pre-existing IgM explains the requirement for C1q in antibody responses (4). This knock-in mouse strain carries a mutation in the IgM heavy chain gene resulting in a serine instead of a proline at amino acid position 436 in the third constant domain (27). This mutation led to disruption of the C1q binding site and resulted in inability of the IgM to activate complement (4, 27). To our surprise, Cµ13 mice had antibody levels which did not significantly differ from those in WT mice after immunization with SRBC or keyhole limpet hemocyanine (KLH) (4). Other known activators of C1q, including specific intracellular adhesion molecule-grabbing non-integrin R1 (SIGN-R1), serum amyloid P component (SAP), and C-reactive protein (CRP), also failed to explain the requirement for complement (4).

The lack of a clear effect on antibody responses in Cµ13 mice left open the question of how to explain the crucial role for complement, in particular C1q, in primary antibody responses. Still puzzled by this conundrum, we decided to re-investigate the antibody response in Cμ13 mice, changing some of the experimental parameters which we thought would facilitate detection of a difference between Cμ13 and WT mice. Different genetic backgrounds may modify the phenotype observed in mice genetically modified on one specific gene (28). Therefore, both the Cμ13 strain backcrossed to BALB/c (BALB.Cμ13) used previously, and an additional Cμ13 strain, backcrossed to C57BL/6 (B6.Cμ13), were tested. To facilitate detection of very small differences in antibody responses, we used littermate controls instead of separate WT BALB/c or C57BL/6 strains in most experiments. Finally, the influence of complement on antibody responses is more pronounced with lower antigen doses (6, 7, 10, 13, 29, 30). Therefore, a very low dose of SRBC was used both for priming and boosting in the current study. In addition to determining antibody responses, we went more in depth to study B cell activation, including differentiation into germinal center (GC) B cells and antibody-secreting effector cells (ASC), as well as the role of pre-immune and antigen-specific IgM for complement opsonization and clearance of the antigen.

## Materials and methods

### Mice

C57BL/6 mice were originally obtained from Taconic Biosciences Inc (Hudson, NY, USA) and BALB/c mice from Bommice (Ry, Denmark). The Cµ13 mice were generated by us (4) and backcrossed for 12 generations onto BALB/c (BALB.Cµ13) or C56BL/6 (B6.Cµ13) background. BALB.Cµ13^wt/mut^ or B6.Cµ13^wt/mut^ heterozygous mice were bred to yield litters of Cµ13^mut/mut^ mice and Cµ13^wt/wt^ littermate controls, either on BALB/c or C57BL/6 background. Where indicated in the figure legends, mice bred from homozygous B6.Cµ13^mut/mut^ or BALB.Cµ13^mut/mut^ breeders and WT controls from WT homozygous breeders were also included. *Cr2*-deficient mice were originally obtained from Dr Hector Molina (12) and backcrossed for 10 generations onto the BALB/c background (BALB.Cr2^-/-^). C3-deficient mice on C57BL/6 background were obtained from Jackson Laboratories, Bar Harbour, ME, USA (B6.C3^-/-^). The mice were age- and sex-matched within each experiment, and both female and male mice were used in all experiments. All mice were bred and maintained in the animal facilities at the National Veterinary Institute (Uppsala, Sweden). All animal studies were approved by the Uppsala Animal Research Ethics Committee (permit number 5.8.18-02583/2018).

### Genotyping of Cµ13 mice

The BALB.Cµ13 and B6.Cµ13 mice were screened for presence of the mutant and WT alleles by PCR on crude lysate from ear biopsies. Briefly, 1 µl of crude biopsy lysate was added to 10 µl of DreamTaq Green PCR Master Mix (2x) (Thermo Scientific) together with 1 µl each of forward primer CU13F (5′-AGGAGCCTCTGTAAGGAGTC-3′) and reverse primer CU13R (5′-TGGGTCTTGGTACCAAGAGA-3′) and 7 µl nuclease free water (4). The PCR reaction was carried out on a thermocycler (Applied Biosystems/ThermoFisher Scientific) (30 s at 95°C; 35 cycles of 30 s at 95°C, 30 s at 52°C, and 1 min at 72°C; 10 min at 72°C). The PCR products were subjected to agarose gel electrophoresis to determine band size (WT band at 200 bp; mutant band at 360 bp).

### Antigen preparation

SRBC in sterile Alsever’s solution were purchased from the National Veterinary Institute (Håtunaholm, Sweden) and stored at 4°C. Before use, cells were washed three times in 10 ml PBS. The SRBC were diluted in PBS to the indicated concentration for immunization or ELISA.

### Immunization and blood sampling

SRBC were prepared as described above and diluted in PBS to 5x10^6^ or 5x10^8^ cells/ml for immunization as indicated. 100 µl was injected intravenously (i.v.) into either one of the lateral tail veins at day 0 and in some experiments mice were boosted at day 21 or 70. Blood was drawn from the tail artery at day 5, 21, 35, and 49 post-primary immunization. Serum was collected and stored at -20°C until further analysis.

### ELISA for IgM- and IgG-anti SRBC

The ELISA was performed as described previously (31), with a few modifications. Briefly, high-binding ELISA plates (Sarstedt) were pre-treated with poly-L-lysin (Sigma-Aldrich), coated with SRBC, and fixed in glutaraldehyde (Sigma-Aldrich). The plates were blocked in PBS 5% dry milk, 0.02% NaN_3_ and incubated at 4°C overnight or at room temperature for 2 hours. Sera were serially diluted (5-fold in four steps, starting at 1:25) in PBS (0.05% Tween 0.25% dry milk, 0.02% NaN_3_), added in duplicate and incubated overnight at 4°C. IgM-anti SRBC was detected with polycloncal goat-anti mouse IgM conjugated to alkaline phosphatase (Jackson ImmunoResearch Laboratories). IgG-anti SRBC was detected with polycloncal goat-anti mouse IgG conjugated to alkaline phosphatase (Jackson ImmunoResearch Laboratories). The plates were developed with p-nitro-phenylphosphate substrate (Sigma-Aldrich) and absorbance measured at 405 nm. After subtracting the blanks and averaging the duplicates, the OD_405_ values from the indicated dilutions were used for analysis.

### Antibodies

For flow cytometry, we used fluorochrome- or biotin-labeled antibodies against B220 (clone RA3–6B2), CD1d (clone 1B1), CD16/CD46 (Fc block; clone 2.4G2), CD19 (clone 1D3), CD21/CD35 (clone 7G6), CD23 (clone B3B4), CD35 (clone 8C12), CD38 (clone 90), CD95 (clone Jo2), CD138 (clone 281-2), GL-7 (clone GL-7), IgD (clone 11–26c.2a), IgM (clone R6–60.2), IgM^a^ (clone DS-1), IgM^b^ (clone AF6-78), and C3 split products (clone RmC11H9); all were purchased from BD Biosciences, Biolegend, eBioscience, or Cedarlane Laboratories. Rabbit polyclonal IgG anti-SRBC was prepared in-house (26). Staining was done in FACS buffer (PBS 2% FBS; Sigma Aldrich) or, when two or more Brilliant Violet™ antibody conjugates were used, in Brilliant Stain Buffer (BD Biosciences). All antibodies were used at ≤0.5 µg per 1x10^6^ cells.

### Analysis of complement opsonization of SRBC

For analysis of complement opsonization of SRBC, 10x to 5x^7^ SRBC was administered to B6.Cµ13^mut/mut^, B6.Cµ13^wt/wt^, B6.C3^-/-^, and B6.C1q^-/-^ mice, naïve or at 21 days post-immunization with 5x10^5^ SRBC. Approximately 100 µl of whole blood was collected at 1 minutes after administration of the SRBC, immediately mixed with 1 µl lepirudin (50 mg/ml; Refludan, Calgene AB) and stained for B cells (B220), SRBC, and C3 split products. The samples were incubated on ice in the dark for 30 minutes, washed once and subsequently resuspended in FACS buffer. Data were acquired using a BD LSRFortessa or a BD FACSAria III (both BD Biosciences). After exclusion of doublets, SRBC were gated out and assessed for deposition of C3 split products (complement opsonization). The data was analyzed using FlowJo v10.7.1 Software (BD Life Sciences) for macOS.

### Flow cytometry analysis of splenic B cells

Spleens from immunized mice were collected post mortem. Single-cell suspensions were prepared by gently mashing the spleen and passing the cell suspension through a 70 µm filter. ACK buffer (0.15 M NH4Cl, 0.1 mM EDTA and 1.0 M KHCO3) was used to lyse splenic erythrocytes. The cells were finally suspended in FACS buffer at 10x10^6^ cells/ml. 100 µl of cell suspension was stained for 30 minutes on ice in the dark. The cells were washed once and subsequently resuspended in FACS buffer before data acquisition on a BD LSRFortessa or a BD FACSAria III. Flow cytometry data was analyzed using FlowJo v10.7.1 Software for macOS. After exclusion of doublets, lymphocytes were gated based on forward and side scatter properties. Marginal zone (MZ) and follicular (FO) B cells were gated from the lymphocyte population as B220^+^ >> CD1d^hi^CD23^lo^ (MZ B cells) or CD1d^lo^CD23^hi^ (FO B cells) and the surface expression of different markers on either population was evaluated as median fluorescent intensity (MFI). Antigen-activated B cells were gated from the lymphocyte population as B220^lo/+^IgD^-^, antibody-secreting cells (ASCs, including both plasmablasts/early plasma cells and bona fide plasma cells) as B220^lo/+^IgD^-^CD138^hi^, and GC B cells as B220^+^IgD^-^CD138^-^CD38^lo^CD95^+^GL7^+^. In addition, presence or absence of surface IgM (i.e. unswitched and class-switched B cells, respectively) was evaluated. Gating strategies are shown in **Supplementary Figure S1**. To investigate frequency of mutant (Cµ13^mut^; IgM^a^) and WT (Cµ13^wt^; IgM^b^) B cells in heterozygous mice, cell suspensions were stained for IgM^a^ and IgM^b^, respectively, in addition to the markers described above.

### Statistical analysis

Data in all figures are pooled from 2-3 independent experiments. In figures with bar graphs and symbols, each symbol represents an individual mouse. Normally distributed data was analyzed by unpaired two-tailed Student’s t-test (two groups) or a one-way ANOVA with Tukey’s multiple comparison test (more than two groups). For data that did not follow a Gaussian distribution, an unpaired Mann Whitney test (two groups) or a Kruskal-Wallis test with Dunn’s multiple comparison test (more than two groups) was used. Statistical analyses were performed using Prism 9 (GraphPad Software, Inc.). All data are presented as mean ± SEM. A p-value <0.05 was considered significant.

* or ‡, p<0.05; ** or ‡‡, p<0.01; *** or ‡‡‡, p<0.001; **** or ‡‡‡‡, p<0.0001. Comparisons that did not reach statistical significance (p≥0.05) are not denoted in the figures, i.e., no symbol (e.g., asterisk) corresponds to no statistically significant difference.

## Results

### Minor differences in peripheral B cell populations of Cµ13 mice

The peripheral B cell compartment in B6.Cµ13 mutant mice, BALB.Cµ13 mutant mice, and the respective WT controls (littermates or bred separately), was characterized by flow cytometry (**Supplementary Figure S2**). The analysis revealed few differences between the strains. However, we confirmed the previous demonstration of an increase in MZ B cells, with a corresponding reduction of FO B cells, in BALB.Cμ13 mutant mice (4). Surprisingly, this difference was not seen in B6.Cμ13 mutants, here analyzed for the first time. Moreover, the expression of CR1 (CD35) on MZ B cells was enhanced in both Cμ13 mutant strains.

### Endogenous complement-activating IgM enhances secondary, but not early primary, IgG responses to SRBC

**Figure 1:**
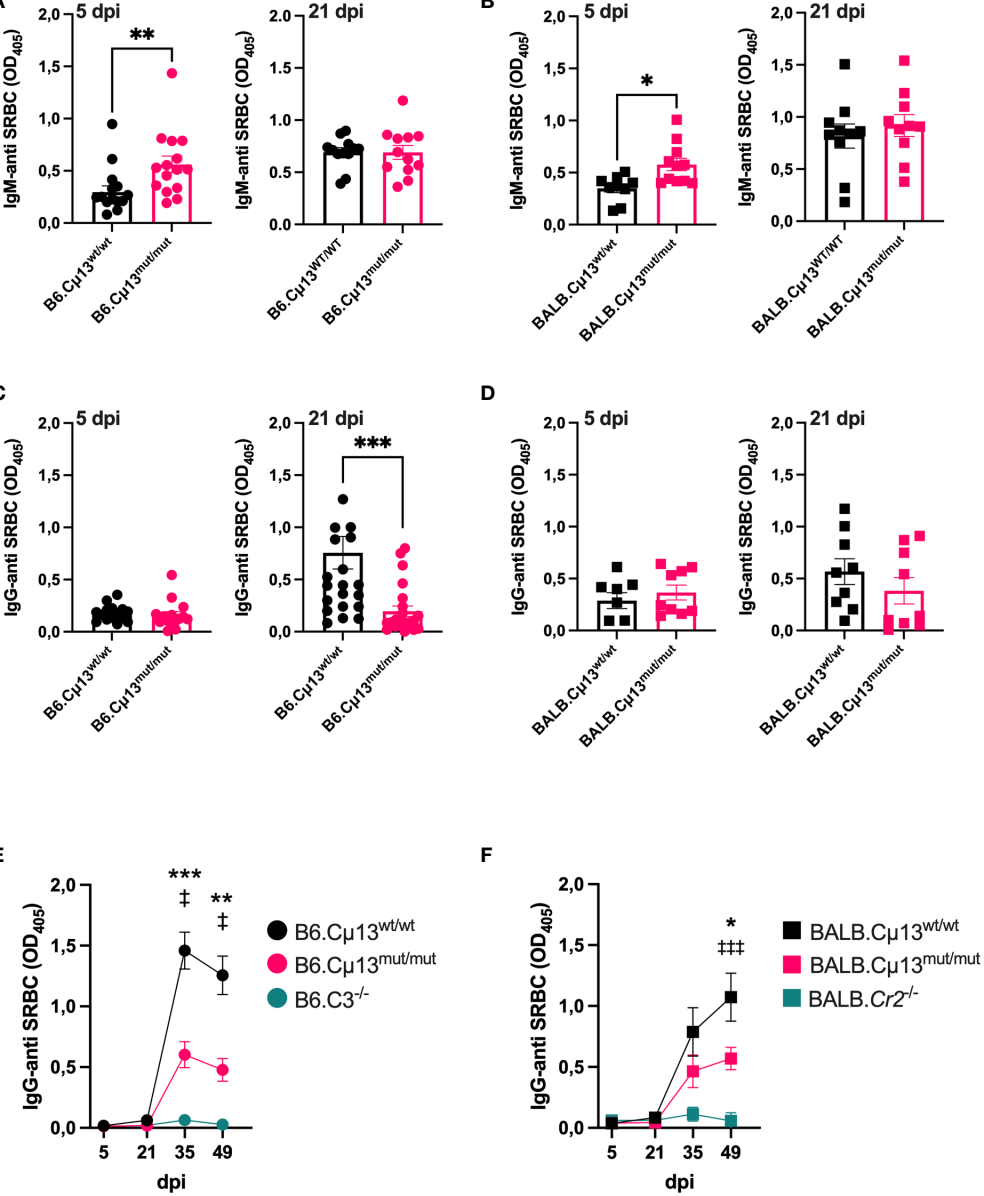
Complement activating IgM is required for robust secondary antibody responses to SRBC. Cµ13^mut/mut^ mice on both C57BL/6 [B6.Cµ13^mut/mut^; **(A)** and **(C)**] and BALB/c [BALB.Cµ13^mut/mut^; **(B, D)**], together with their respective WT littermate controls (Cµ13^wt/wt^) and either C3 knockout (B6.C3^-/-^) or Cr2 knockout (BALB.*Cr2* ^-/-^) mice were immunized iv with 5x10^5^ SRBC in 100 µl PBS on day 0 and boosted with the same dose on day 19/21. The mice were bled on days 5, 21, 35, and 49 post-primary immunization, and serum IgM and IgG specific for SRBC were detected by ELISA. **(A, B)** Serum IgM-anti SRBC on day 5 (left) and day 21 (right) post-immunization in mice on C57BL/6 **(A)** and BALB/c **(B)** background. The sera were diluted 1:25. **(C, D)** Serum IgG-anti SRBC on day 5 (left) and day 21 (right) post-immunization in mice on C57BL/6 **(C)** and BALB/c **(D)** background. The sera were diluted 1:25. **(E, F)** IgG-anti SRBC on days 5, 21, 35, and 49 post-immunization in mice on C57BL/6 **(E)** and BALB/c **(D)** background. Sera were diluted 1:625. * denotes statistical difference between Cµ13^wt/wt^ and Cµ13^mut/mut^ mice; ‡ denotes statistical difference between Cµ13^wt/wt^ and either B6.C3^-/-^ or BALB.Cr2^-/-^ mice. Data are pooled from 2 [**(A)** left panel, **(B, C)** left panel, and **(D)**] or 3 [**(A)** right panel, **(C)** right panel, **(E)**, and **(F)**] independent experiments; total n=2 for B6.C3^-/-^, n=5 for BALB/c.*Cr2^-/-^*, and n=10-24 for all other groups.

To test our hypothesis that pre-immune endogenous IgM can enhance antibody responses to large particulate antigen by activation of the classical complement pathway, we immunized B6.Cµ13^mut/mut^ mice, littermate WT controls (B6.Cµ13^wt/wt^), and B6.C3^-/-^ mice with 5x10^5^ SRBC i.v. on day 0. The mice were boosted with the same dose on day 21. To compare with previous findings (4), we also included BALB.Cµ13^mut/mut^ mice, littermate controls (BALB.Cµ13^wt/wt^), and BALB.Cr2^-/-^ mice. B6.C3^-/-^ (32) and BALB.Cr2^-/-^ mice (4, 13), known to have very poor IgG responses to SRBC, were included as negative controls in analyses of the IgG responses. Blood samples were collected on days 5, 21, 35, and 49 post-primary immunization, and the sera were analyzed by ELISA for SRBC-specific IgM (days 5 and 21) and IgG (all days). Similar to previous findings from our lab (4), the early IgM response on day 5 was higher in the Cµ13^mut/mut^ mice compared to Cµ13^wt/wt^ littermate controls (**Figures 1A, B**, left). This was seen in mice on both B6 and BALB/c background. On day 21, the IgM response was similar in mutant and control mice (**Figures 1A, B**, right). The primary IgG response (days 5 and 21) was very low in all mice, but we did observe a decrease in the B6.Cµ13^mut/mut^ but not in the BALB.Cµ13^mut/mut^ mice on day 21 (**Figures 1C, D**). At 2 weeks post-boost (day 35), IgG-anti SRBC levels had increased substantially in all mice except B6.C3^-/-^ and BALB.Cr2^-/-^. At this timepoint, B6.Cµ13^mut/mut^ mice had significantly lower levels of SRBC-specific IgG than B6.Cµ13^wt/wt^ control mice (**Figure 1E**). This difference was sustained through day 49 (**Figure 1E**). Similarly, BALB.Cµ13^mut/mut^ mice tended to have less IgG-anti SRBC on days 21 (at serum dilution 1:25) (**Figure 1D**, right) and 35 (**Figure 1F**). This difference reached statistical significance at day 49 post-primary immunization (**Figure 1F**). These findings demonstrate that activation of the classical complement pathway by pre-existing (natural) IgM does not enhance early primary antibody responses. However, activation of the classical complement pathway by IgM is important for optimal secondary antibody responses, an effect probably mediated by specific IgM induced after priming. Importantly, this observation does not explain the entire effect of complement on antibody responses because C3^-/-^ and Cr2^-/-^ mice have a much more dramatically reduced response than the Cµ13^mut/mut^ mice (**Figures 1E, F**; **Supplementary Figure S3**).

### B cells with membrane-bound IgM incapable of activating complement are less prone to differentiate into antibody-secreting cells than WT B cells

**Figure 2:**
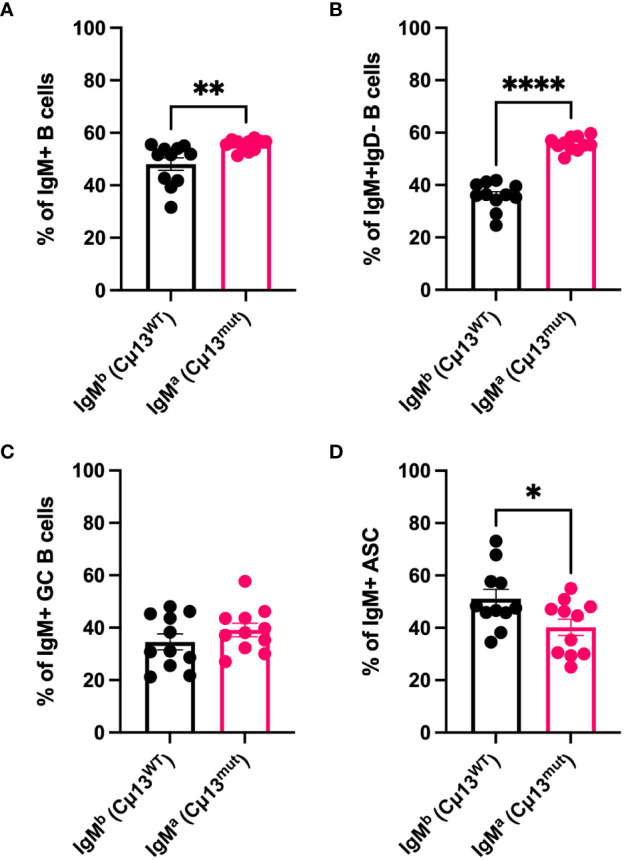
B cells with membrane-bound IgM incapable of activating complement are less prone to differentiate into antibody-secreting cells than WT B cells. B6 mice heterozygous for the mutant Cµ13 allele (B6.Cµ13^WT/mut^ mice) were immunized i.v. with 5x10^5^ SRBC in 100 µl PBS on day 0. Spleens were harvested on day 5 and analyzed by flow cytometry for WT (IgM^b^; Cµ13^WT^) and mutant (IgM^a^; Cµ13^mut^) B cells within the ASC (IgD^-^CD138^hi^) and GC (IgD^-^CD138^-^CD38^lo^CD95^hi^) compartments. **(A, B)** Frequency of WT and mutant B cells amongst total IgM+ **(A)** and antigen-activated (IgD-) IgM+ **(B)** B cells. **(C)** Frequency of WT and mutant GC B cells of total IgM+ GC B cells. **(D)** Frequency of WT and mutant ASCs of total IgM+ ASCs. Data are pooled from 2 independent experiments; total n=11; ASC, antibody-secreting cells; GC, germinal center. *, p<0.05; **, p<0.01; ****, p<0.0001.

To understand why complement-activating IgM contributes to increased secondary IgG responses we first wanted to investigate whether B cells carrying mutant IgM have a disadvantage compared to WT B cells in terms of antigen-activation and differentiation into GC B cells and ASCs. Not only secretory, but also membrane-bound IgM can activate complement by binding C1q (33, 34), thus potentially decreasing the activation threshold by crosslinking the B cell receptor and CR2 (CD21) of the BCR coreceptor (35–37). We took advantage of the fact that the knock-in cassette containing the mutant allele is of IgM^a^ allotype (4) and that WT C57BL/6 IgM is of the b allotype (IgM^b^). Consequently, owing to allelic exclusion, 50% of B cells in the heterozygous (Cµ13^mut/mut^ x Cµ13^wt/wt^) F1 mice (i.e. B6.Cµ13^wt/mut^) would be IgM^a^ (Cµ13^mut^) and 50% IgM^b^ (Cµ13^wt^). We hypothesized that, within the same individual, mutant B cells would have a disadvantage compared to WT B cells in terms of receiving signals for activation and differentiation. To test this, B6.Cµ13^wt/mut^ mice were immunized with 5x10^5^ SRBC on day 0 and spleens harvested on day 5. Flow cytometry analysis of the immunized mice revealed a roughly equal but with a slight advantage for mutant (45/55; WT/mutant) distribution of the allotypes within the IgM^+^ B cell compartment (**Figure 2A**). Zooming in on the antigen-activated (IgD^-^) B cells (**Figure 2B**), the mutant B cells still demonstrated a higher frequency, thus showing no activation disadvantage compared to WT B cells. Despite this discrepancy, the relative contribution of WT and mutant B cells to the IgM^+^ GC B cell pool was similar (**Figure 2C**; for gating strategy see **Supplementary Figure S1A**). Interestingly, WT B cells made up a larger fraction of IgM^+^ ASCs than did mutant B cells, thus demonstrating a disadvantage for the latter in the ability to differentiate into effector cells (**Figure 2D**). The specificity of the antibodies used to detect IgM^a^ and IgM^b^, respectively, was confirmed testing them on WT C57BL/6 mice and B6.Cµ13 mutant mice (**Supplementary Figure S4**).

### Complement-activating IgM promotes differentiation into antibody secreting cells but not germinal center B cells in recall responses

As we demonstrate in **Figure 2**, IgM^+^ B cells carrying the mutant allele are less likely to fully differentiate into ASCs than WT B cells when competing in the same individual. However, since the Igh^a^ allele is present only in the knock-in cassette containing the constant µ chain (4), that system is limited to the study of unswitched (IgM^+^) B cells. We wanted to determine whether induction into class-switched ASC and/or germinal center B cells as well as recall of memory B cells (both unswitched and switched) was impaired when IgM was incapable of complement activation. To this end, we immunized B6.Cµ13^mut/mut^ mice, littermate WT controls (B6.Cµ13^wt/wt^), and B6.C3^-/-^ mice with 5x10^5^ SRBC i.v. on day 0 and boosted with the same dose on day 70. Spleens were harvested on days 5 and 10 (early GC and ASC response), and day 75 (5 days post-boost; recall response, both GC B cells and ASC) and were analyzed by flow cytometry (antigen-activated cells; frequency of GC B cells and ASCs; the gating strategy is shown in **Supplementary Figure S1A**).

**Figure 3:**
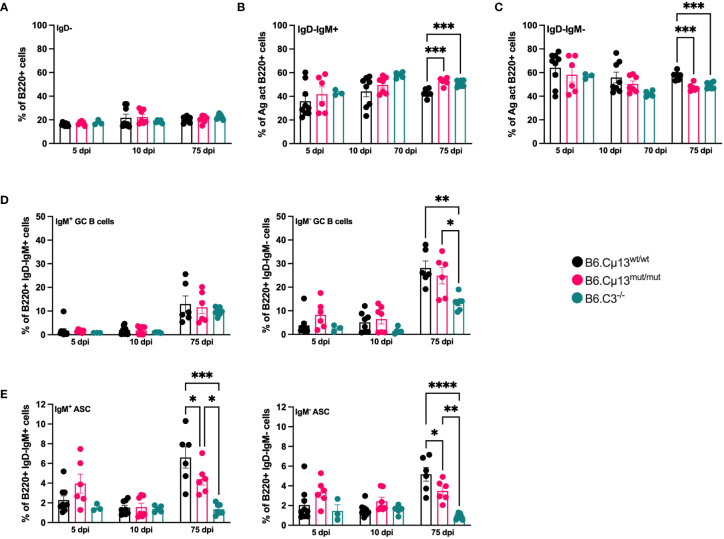
Complement-activating IgM is needed for differentiation into ASCs but not GC B cells. B6.Cµ13^mut/mut^ and WT littermate controls (B6.Cµ13^wt/wt^), along with B6.C3^-/-^ mice were immunized i.v. with 5x10^5^ SRBC in 100 µl PBS on day 0 and boosted with the same dose on day 70. Spleens were harvested on day 5 and 10 post-primary immunization, and five days after boost (day 75), and analyzed by flow cytometry for B cell activation and differentiation into ASCs and GC B cells. **(A)** Frequency of antigen-activated (IgD-) B cells. **(B, C)** Frequency of IgM+ (unswitched; **(B)**) and IgM- (class-switched; **(C)**) antigen-activated B cells. **(D)** Frequency of GC B cells out of antigen-activated (IgD-) IgM^+^ (left) and IgM^-^ (class-switched; right) B cells. **(E)** Frequency of ASCs out of antigen-activated (IgD^-^) IgM^+^ (left) and IgM^-^ (class-switched; right) B cells. Data are pooled from 2 independent experiments; total n=3-9. *, p<0.05; **, p<0.01; ***, p<0.001; ****, p<0.0001.

Neither complete absence of C3 nor the incapability of IgM to activate the classical pathway had any impact on the frequency of total antigen-activated (IgD^-^) B cells (**Figure 3A**). However, WT mice had a greater propensity to quickly class-switch after the boost, as reflected by a lower frequency of IgM^+^IgD^-^ B cells (**Figure 3B**) and a corresponding higher frequency of IgM^-^IgD^-^ B cells (**Figure 3C**) on day 75 compared to both B6.Cµ13^mut/mut^ and B6.C3^-/-^ mice.

The frequency of both unswitched (IgM^+^) and switched (IgM^-^) germinal center B cell was similar in all mice and at all time points except on day 75. Here, the frequency of switched GC B cells was significantly lower in C3^-/-^ mice than in the two other strains (**Figure 3D**, right). The differentiation into both unswitched (IgM^+^) and class-switched (IgM^-^) ASCs was similar in all mice during the early primary response (days 5 and 10; **Figure 3E**). However, at five days after boost (day 75), the frequency of both unswitched and switched ASCs from B6.Cµ13^mut/mut^ mice was significantly lower than in WT littermate controls (**Figure 3E**). B6.C3^-/-^ mice demonstrated a dramatically diminished capacity to rapidly form ASC following primary as well as secondary immunization, compared to both WT or Cµ13 mutant mice (**Figure 3E**). Thus, B6.Cµ13^wt/wt^ have an advantage over B6.Cµ13^mut/mut^ mice which have an advantage over B6.C3^-/-^ mice in differentiation into ASC after boost.

### Complement opsonization of large particulate antigen is dependent on antigen-specific, but not pre-immune, IgM

**Figure 4:**
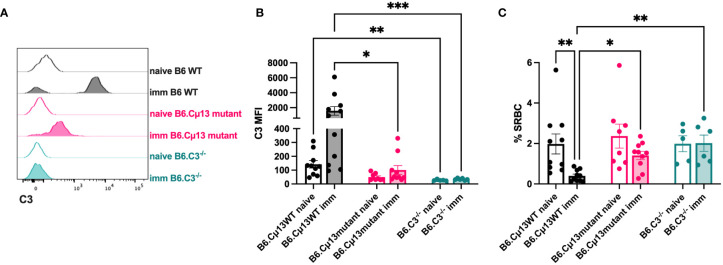
Complement opsonization and clearance of the antigen is enhanced by antigen-specific, but not natural, IgM. Cµ13 mutant mice and C57BL/6 WT control mice were immunized iv with 5x10^5^ SRBC in 100 µl PBS on day 0. The immunized mice, together with naive control mice, were given 5x10^7^ SRBC iv in 100 µl PBS on day 21 and subsequently bled after 1 minute. The blood was analyzed by flow cytometry for deposition of C3 split products on the SRBC and frequency of SRBC. **(A)** Histograms showing C3 deposition on SRBC from naive or immunized mice. **(B)** MFI of C3 split products on SRBC after 1 minute. **(C)** Frequency of SRBC (out of singlets) from naive and immunized mice. Data are pooled from 2 independent experiments; total n=5-11. This experiment includes both B6.Cμ13mut/mut mice with B6.Cµ13wt/wt littermate controls and mice bred from homozygous B6.Cµ13mut/mut breeders with C57BL/6 WT controls from WT homozygous breeders. *, p<0.05; **, p<0.01; ***, p<0.001.

Because secreted IgM is present in the circulation at the time of any antigen encounter (i.e. natural IgM), we lastly explored whether also such pre-immune IgM contributes to antibody responses by its complement-activating function. Complement opsonization of antigen very early, even immediately, could contribute to increased transport by CR1/CR2-expressing MZ B cells into B cell follicles and increased deposition of immune complexes on follicular dendritic cells (38–40). Moreover, it may lead to increased crosslinking of BCR and CR2 of the B cell co-receptor complex known to lower the threshold for B cell activation (35–37). We hypothesized that pre-immune (natural) IgM in the circulation can recognize and bind to large particulate antigen administered i.v., thus activating the classical complement pathway resulting in a stronger antibody response to the immunogen. To test this, we administered 5x10^7^ SRBC i.v. to naïve or immunized (day 21 post-immunization with 5x10^5^ SRBC i.v.) B6.Cµ13 mutant mice, B6 WT controls (littermates or bred separately), and B6.C3^-/-^ mice and bled them after 1 minute. The blood was analyzed by flow cytometry for deposition of C3 split products on the SRBC (**Figures 4A, B**) as well as for SRBC-presence in the circulation after 1 minute (to indicate antigen clearance) (**Figure 4C**). Naïve mice showed little (B6 WT) or no (B6.Cµ13 mutant) C3-opsonization of SRBC above that from B6.C3^-/-^ negative control mice (**Figures 4A, B**). There was no difference between naïve Cµ13 mutant and WT mice, indicating that pre-immune IgM does not play a major role in complement activation and opsonization of large particulate antigen. However, since the difference in antibody response between B6.Cµ13^mut/mut^ and B6.Cµ13^wt/wt^ mice is most pronounced during the secondary response, we also asked whether antigen-specific IgM present at the time of boost (day 21) contributes to C3-deposition on SRBC. As expected, in immunized WT mice we found a much higher degree of C3 deposition on SRBC than in naïve animals (**Figures 4A, B**). In B6.Cµ13 mutant mice the opsonization of SRBC was significantly less dense than in the WT controls, and did not increase significantly over naïve mutant mice (**Figures 4A, B**). In addition, we looked at the amount of SRBC present in peripheral blood 1 minute after injection, with a higher frequency of SRBC indicating a slower clearance rate from the circulation and vice versa. In WT mice, the clearance is significantly increased at day 21 post-immunization compared to naïve controls (**Figure 4C**). Neither Cµ13 mutant nor B6.C3^-/-^ mice increase the clearance rate after immunization, and both demonstrate a higher frequency of SRBC in peripheral blood of immunized mice compared to WT controls (**Figure 4C**). These data indicate that antigen-specific IgM already present can contribute to complement activation and subsequent clearance of immune complexes upon secondary antigen encounter whereas complement activation by pre-immune IgM appears to have little effect.

## Discussion

The major purpose of the present study was to determine whether natural, pre-existing IgM could explain the role for complement, in particular C1q, in primary antibody responses. The idea that pre-immune IgM would be able to activate complement is based on the assumption that the 10 antigen binding sites on the IgM molecule, although each would be of low affinity, would make the IgM “sticky” enough to bind SRBC and activate C1q. Two observations supported this hypothesis: (i) mice lacking secretory IgM have been reported to have an impaired antibody response (22, 41) and this defect could be rescued by transfer of IgM from naïve mice (22) and (ii) specific IgM, co-administered with antigen, enhances the antibody response to the antigen provided the IgM can activate complement (25, 26).

In a previous report, we tested this hypothesis by priming BALB.Cµ13 mutant mice with 5x10^5^, 5x10^6^, 5x10^7^, or 5x10^8^ SRBC and boosting with 5x10^6^ SRBC. No consistent reduction of the antibody response in the mutant mice compared to WT BALB/c mice was observed, although an impairment was observed in odd experiments (4). We here re-investigated the antibody response in Cµ13 mutant mice and extended the analysis to B cell differentiation and opsonization of antigen. To facilitate detection of any differences, we primed and boosted with a very low antigen dose (5x10^5^ SRBC), used littermate controls, and included mutant mice back-crossed also to the C57BL/6 strain. In analogy with our previous observations, there was no impairment of early IgM or IgG responses in Cμ13 mutant mice. Thus, complement-activating natural IgM, present in naive mice at the time of primary immunization, does not seem to enhance early responses to SRBC. In summary, neither the present nor the previous (4) observations support the idea that complement activation by natural IgM explains why C1q is required for robust primary antibody responses and the explanation for this paradox remains enigmatic.

Interestingly, the present experiments do suggest a role for endogenous specific IgM in enhancing late primary as well as secondary IgG responses. The first sign of an impaired antibody response in Cμ13 mutant mice was observed 21 days after priming when the IgG anti-SRBC levels were reduced in one of the mutant strains, B6.Cµ13^mut/mut^. A more profound effect was observed after boosting, when the IgG responses were impaired both in BALB.Cµ13^mut/mut^ and B6.Cµ13^mut/mut^ mice. This reduction was paralleled by reduced levels of switched and unswitched ASC. Moreover, the SRBC administered day 21 after boost were quickly opsonized with complement factors in WT but not in Cμ13 mutant mice, suggesting that complement-activating IgM plays an important role. We cannot exclude that the reduced levels of SRBC-specific IgG observed in Cμ13 mutant mice day 21 (**Figure 1**) contributed to the impaired opsonization. However, given that IgG, unlike IgM, requires aggregation before it can bind C1q and that the IgG levels in WT mice day 21 is very low, IgM is likely to be the major cause of opsonization in our experiment.

The findings suggest that specific WT IgM generated after priming, as well as the IgM produced after boost, act to enhance the secondary antibody response. This would be analogous to the IgM-mediated, complement-dependent enhancement of antibody responses seen after passive administration of IgM anti-SRBC together with low doses of SRBC (23–26, 30, 38). The ability of endogenously produced, antigen-specific complement-activating IgM to enhance antibody responses may be important in a physiological situation. Although the effect could only be revealed using low SRBC doses, this potentiation step may still be biologically relevant because low doses of pathogen is most likely what is encountered during the initial stages of an infection.

These experiments were not designed to address the mechanism behind the ability of specific IgM to enhance antibody responses. Nevertheless, the efficient opsonization of SRBC seen in WT but not mutant mice after boost, is likely to be a first step in the chain of events leading to the enhanced antibody responses induced by IgM. Several mechanisms for how IgM and complement potentiates antibody responses have been discussed. Complement opsonization of antigen enables cross-linking of the BCR and its co-receptor (CD19/CR2/CD81), thus decreasing the threshold for B cell activation (35–37). Whether this also leads to increased antibody responses *in vivo* has been a matter of debate. In murine systems, some investigators report a major role for CR1/CR2 on B cells in generating antibody responses (10, 11) whereas others find a major role for CR1/CR2 expressed on follicular dendritic cells (42–45). In a recent study, it was shown that CR1/CR2 expression on human primary B cells did not lead to increased antibody production *in vitro* (46). A not mutually exclusive possibility is that IgM-antigen-complement complexes are transported into B cell follicles by CR1/CR2-expressing MZ B cells followed by increased capture and presentation of antigen on CR1/CR2-expressing follicular dendritic cells (38–40, 45). The latter scenario would lead to an increased concentration of antigen in B cell follicles and thereby more efficient stimulation of the immune system. The present report implies that a direct effect on B cell development is unlikely, because only minor changes in the B cell compartment (increased MZ B cells and decreased FO B cells) of Cμ13 mutant mice were noted. Moreover, these differences were only observed in mutant mice on BALB/c background and the antibody response is less impaired in this strain than in mice on C57BL/6 background. The enhanced induction of antibody responses by IgM-SRBC-complement complexes depends heavily on follicular dendritic cells expressing CR1/CR2, with a minor contribution of CR1/CR2-expressing B cells (45). Therefore, the most likely explanation for the enhancing effect of IgM in antibody responses against SRBC in WT mice is increased capture of the immune complexes by follicular dendritic cells. However, transport of the immune complexes into B cell follicles by MZ B cells as well as increased signaling by co-crosslinking of BCR and its co-receptor (CD19/CR2/CD81) may add to the effect (45). The latter may in fact explain the reduced capacity for ASC differentiation of mutant B cells, as a stronger activation signal promotes this fate decision over entering a GC (47). When responses to IgM-KLH-NP-complement immune complexes were studied, transport by CR1/CR2-expressing MZ B cells seems to play a more pronounced role than deposition of immune complexes on follicular dendritic cells (39).

A very important observation in the present study is that the impairment of IgG responses (**Figures 1E, F**; **Supplementary Figure S3**) or ASC (**Figure 3E**) in Cμ13 mutant mice is not nearly as pronounced as the impairment seen in mice lacking either C3 or CR1/CR2. Therefore, although inability of IgM to activate complement hampers the secondary IgG response, this only partially explains the role of complement in secondary antibody responses. This conclusion is further supported by our previous study in BALB.Cµ13 mutant mice, in which no consistent impairment of the antibody response was seen although responses in Cr2^-/-^ mice immunized in parallel were severely impaired (4). Additional support comes from observations in mice lacking C1q, C3, or CR1/CR2, in which antibody responses to SRBC are strongly impaired to antigen doses which are 10-1000-fold higher than the one (5x10^5^/mouse) used herein (1, 3, 4, 12, 13, 45).

In conclusion, the data presented here suggest that complement activation by natural non-immune IgM does not explain why C1q is important for robust primary antibody responses to antigen (SRBC). However, activation of complement by endogenous specific IgM, produced after immunization, causes opsonization of SRBC and enhancement of late primary as well as secondary IgG responses. Importantly, the impairment of antibody responses in mutant mice is not as pronounced as the impairment seen in C3 or CR1/CR deficient mice, demonstrating that complement activation by specific IgM only partially explains the enhancing effects of complement on antibody responses.

## Data availability statement

The original contributions presented in the study are included in the article/**Supplementary Material**. Further inquiries can be directed to the corresponding authors.

## Ethics statement

The animal study was approved by Uppsala Animal Research Ethics Committee (permit number 5.8.18-02583/2018). The study was conducted in accordance with the local legislation and institutional requirements.

## Author contributions

A-KP: Conceptualization, Formal analysis, Funding acquisition, Investigation, Writing – original draft, Writing – review & editing. AW: Investigation, Writing – review & editing. DA: Investigation, Writing – review & editing. BH: Conceptualization, Funding acquisition, Writing – review & editing.
