# Supplementary material for: Endogenous complement-activating IgM is not required for primary antibody responses but promotes plasma cell differentiation and secondary antibody responses to a large particulate antigen in mice
Source: Front Immunol. 2024 Jan 8;14:1323969. doi: 10.3389/fimmu.2023.1323969 (PMC10800517; doi:10.3389/fimmu.2023.1323969)
Supplement: Supplementary file 1 [file Image_1.pdf]

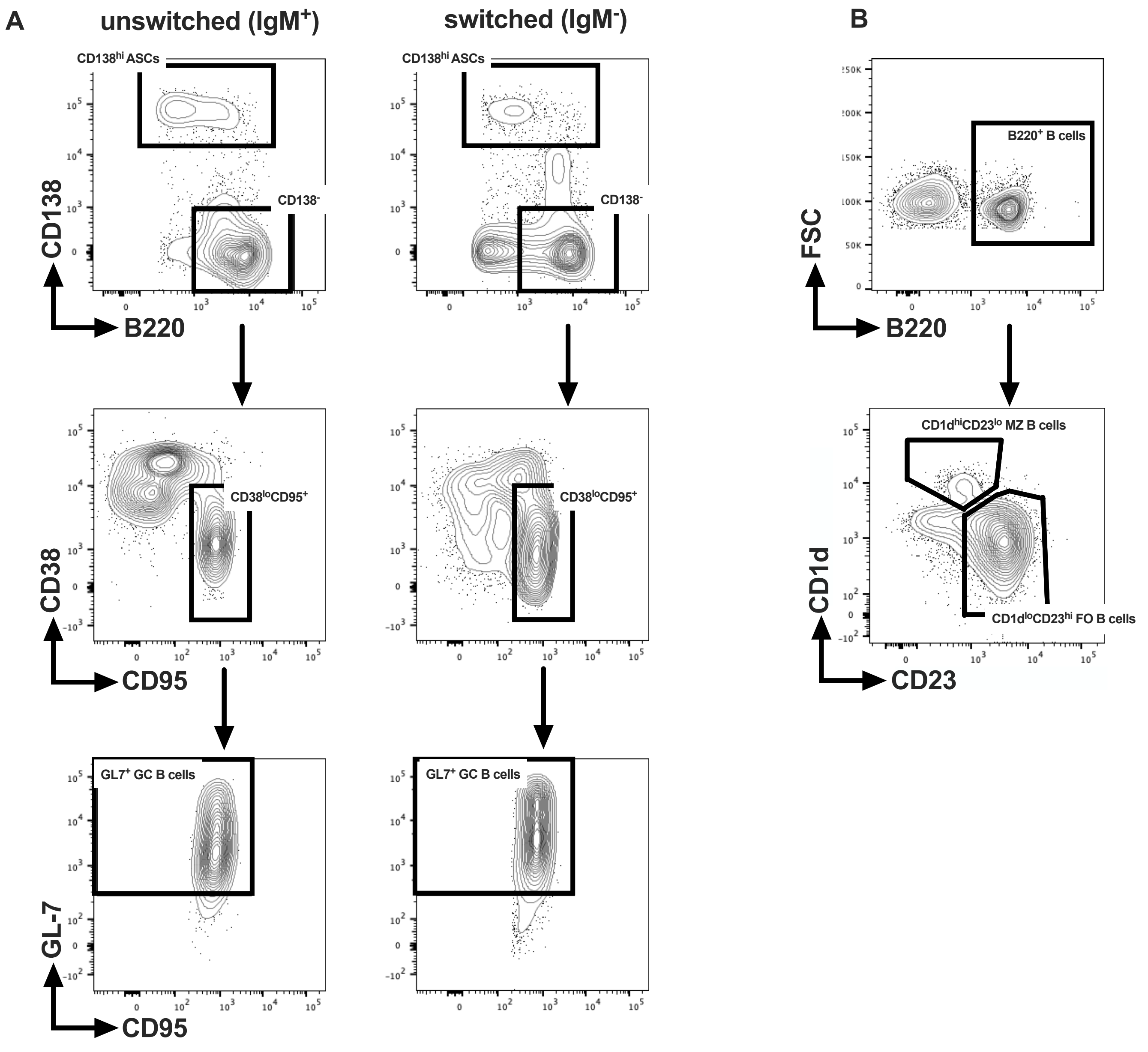


**Supplementary Figure S1. A The gating strategy for ASC and GC B cells.** Splenocytes were first gated on lymphocytes >> singlets >> B220$^{lo/+}$ >> IgD$^{-}$ >> IgM$^{+}$ (left) or IgM$^{-}$ (right). **B The gating strategy for MZ and FO B cells.** Representative plot from one C57BL/6 WT mouse. Splenocytes were first gated on lymphocytes >> singlets.

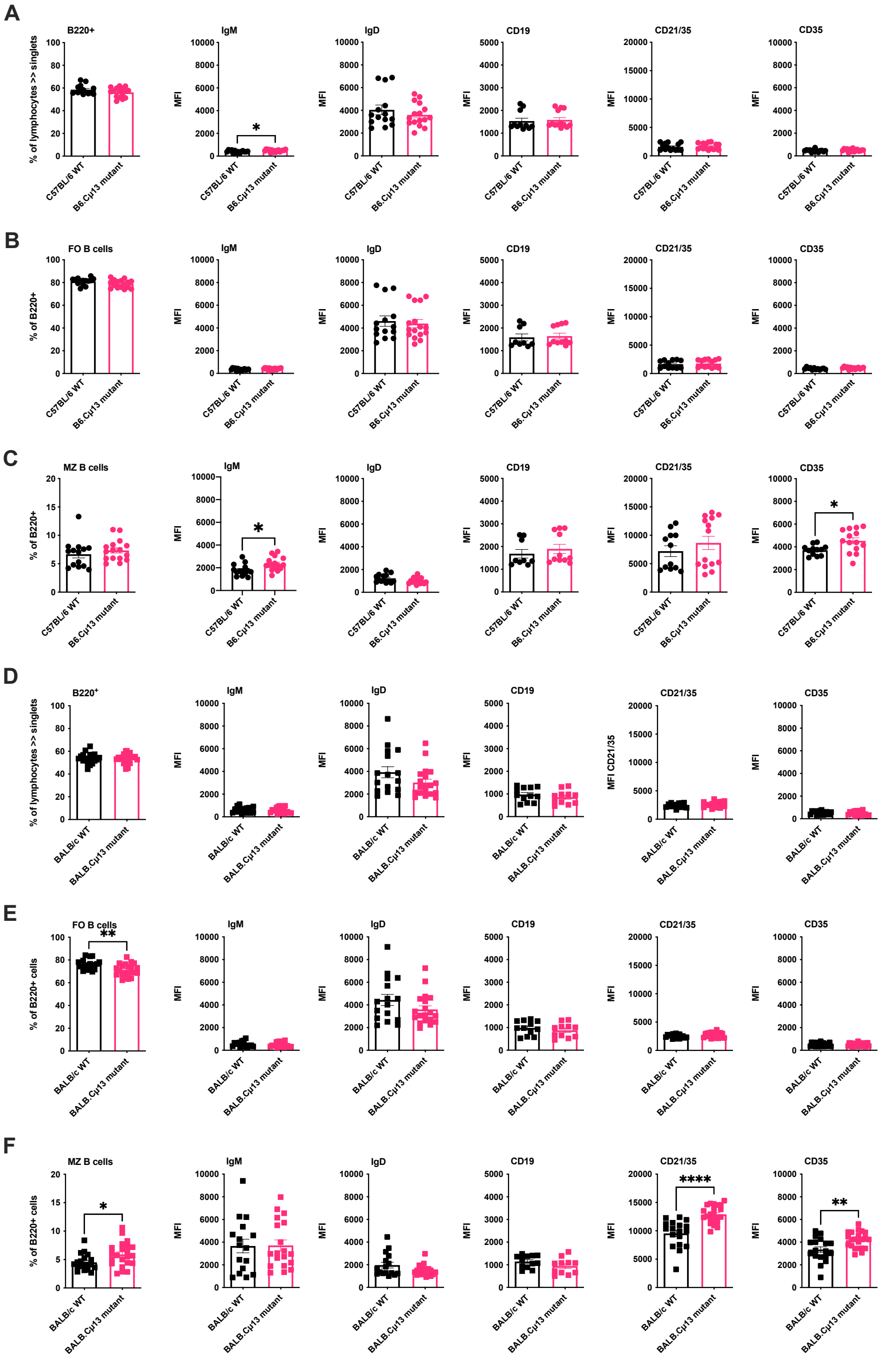


**Supplementary Figure S2. Characterization of the B cell compartment in mice lacking complement-activating IgM. (A)-(C)** Spleens from naive B6.Cµ13$^{mut/mut}$ and WT or WT littermate controls (C57BL/6 or B6.Cµ13$^{wt/wt}$) were analyzed by flow cytometry for different B cell subsets and B cell surface receptor expression. **(A)** Total B cells **(B)** FO B cells **(C)** MZ B cells **(D)-(F)** Spleens from naive BALB.Cµ13$^{mut/mut}$ and WT or WT littermate controls (BALB/c or BALB.Cµ13$^{wt/wt}$) were analyzed by flow cytometry for different B cell subsets and B cell surface receptor expression. **(D)** Total B cells **(E)** FO B cells **(F)** MZ B cells

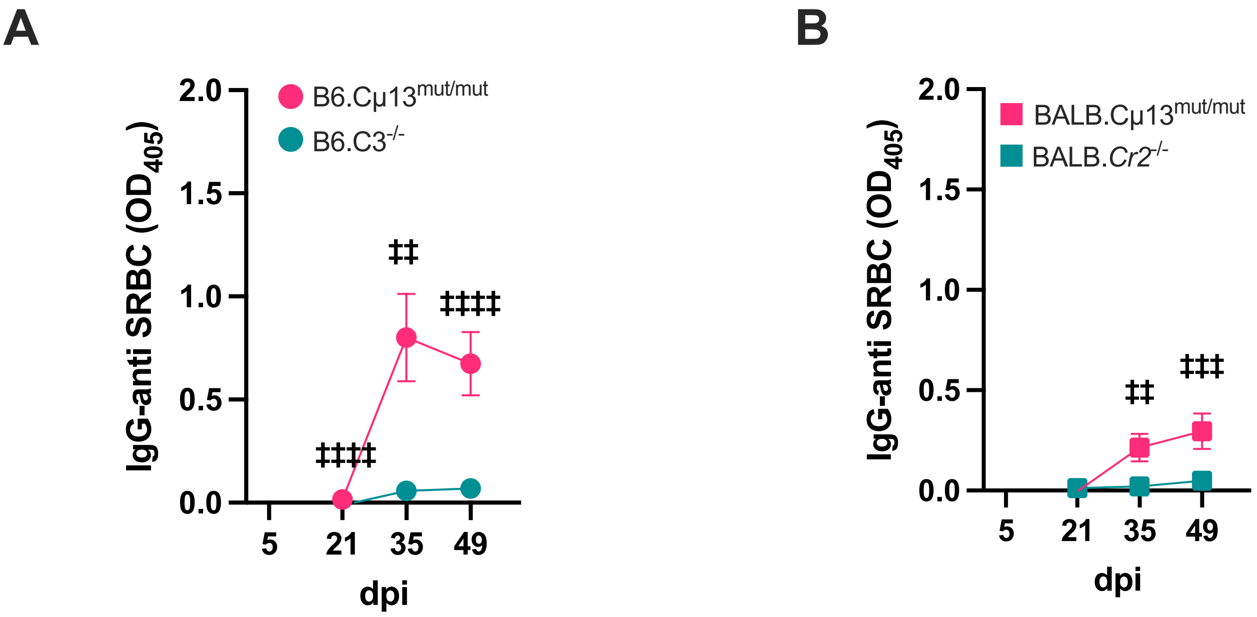


**Supplementary Figure S3. Complement activating IgM is required for robust secondary antibody responses to SRBC.** Cμ13$^{mut/mut}$ mice, either C57BL/6 (B6.Cμ13$^{mut/mut}$; a) or BALB/c (BALB.Cμ13$^{mut/mut}$; b), together with either C3 knockout (B6.C3$^{-/-}$) or Cr2 knockout (BALB.*Cr2*$^{-/-}$) mice, respectively, were immunized iv with 5x10$^{5}$ SRBC in 100 μl PBS on day 0 and boosted with the same dose on day 21. The mice were bled on days 21 (pre-boost), 35, and 49, and serum IgG specific for SRBC was measured by ELISA. **(A)-(B)** IgG-anti SRBC on days 21, 35, and 49 post-immunization in mice on C57BL/6 **(A)** and BALB/c **(B)** background. Sera were diluted 1:625. ‡ denotes statistical difference between Cμ13$^{mut/mut}$ and either B6.C3$^{-/-}$ or BALB.Cr2$^{-/-}$ mice. Data are pooled from 2 independent experiments; total n=9-19. This experiment includes both Cμ13$^{mut/mut}$ mice with Cμ13$^{wt/wt}$ littermate controls and mice bred from homozygous Cμ13$^{mut/mut}$ breeders with WT controls from WT homozygous breeders.

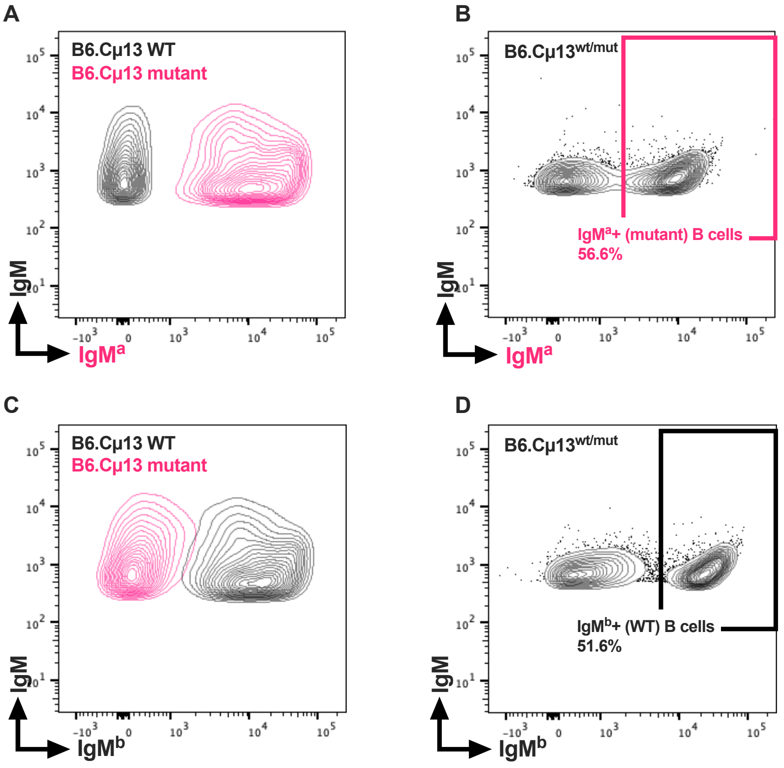


**Supplementary figure S4. B6.Cμ13$^{wt/mut}$ mice have a roughly equal distribution of B cells carrying the WT and mutant alleles.** Spleens from naive B6 mice heterozygous for the mutant Cμ13 allele (B6.Cμ13$^{wt/mut}$ mice) analyzed by flow cytometry for WT (Cμ13$^{WT}$; IgM$^{b}$) and mutant (Cμ13$^{mut}$; IgM$^{a}$) B cells **(A)** Overlay of IgM$^{+}$ B cells from a B6.Cμ13$^{wt/wt}$ (black) and a B6.Cμ13$^{mut/mut}$ (magenta) mouse analyzed for IgM$^{a}$, confirming the specificity of the antibody. **(B)** IgM$^{+}$ B cells from a B6.Cμ13$^{wt/mut}$ mouse analyzed for IgM$^{a}$, demonstrating the presence of the mutant allele in roughly half the B cells. **(C)** Overlay of IgM$^{+}$ B cells from a B6.Cμ13$^{wt/wt}$ (black) and a B6.Cμ13$^{mut/mut}$ (magenta) mouse analyzed for IgM$^{b}$, confirming the specificity of the antibody. **(D)** IgM$^{+}$ B cells from a B6.Cμ13$^{wt/mut}$ mouse analyzed for IgM$^{b}$, demonstrating the presence of the WT allele in roughly half the B cells.
